# A population level study on the determinants of COVID-19 vaccination rates at the U.S. county level

**DOI:** 10.1038/s41598-024-54441-x

**Published:** 2024-02-21

**Authors:** Ensheng Dong, Kristen Nixon, Lauren M. Gardner

**Affiliations:** 1 Department of Civil and Systems Engineering, Johns Hopkins University, Baltimore, MD 21218 USA; 2 Center for Systems Science and Engineering, Johns Hopkins University, Baltimore, MD 21218 USA; 3 Department of Epidemiology, Johns Hopkins Bloomberg School of Public Health, Baltimore, MD 21205 USA

**Keywords:** Infectious diseases, Epidemiology, Health policy, Public health

## Abstract

Multiple COVID-19 vaccines were proven to be safe and effective in curbing severe illness, but despite vaccine availability, vaccination rates were relatively low in the United States (U.S.). To better understand factors associated with low COVID-19 vaccine uptake in the U.S., our study provides a comprehensive, data-driven population-level statistical analysis at the county level. We find that political affiliation, as determined by the proportion of votes received by the Republican candidate in the 2020 presidential election, has the strongest association with our response variable, the percent of the population that received no COVID-19 vaccine. The next strongest association was median household income, which has a negative association. The percentage of Black people and the average number of vehicles per household are positively associated with the percent unvaccinated. In contrast, COVID-19 infection rate, percentage of Latinx people, postsecondary education percentage, median age, and prior non-COVID-19 childhood vaccination coverage are negatively associated with percent unvaccinated. Unlike previous studies, we do not find significant relationships between cable TV news viewership or Twitter misinformation variables with COVID-19 vaccine uptake. These results shed light on some factors that may impact vaccination choice in the U.S. and can be used to target specific populations for educational outreach and vaccine campaign strategies in efforts to increase vaccination uptake.

## Introduction

Vaccines are arguably the most effective tool for combating COVID-19, reducing the number of cases, and more critically, severe illness and hospitalizations, from the disease^1^. One study estimated that the COVID-19 vaccine saved 14.4 million lives globally within one year of its introduction^2^. For the U.S., a study estimated that 240,797 COVID-19 deaths could have been prevented through vaccination from December 12, 2020 to June 30, 2021^3^. As of December 2021, one year since COVID-19 vaccinations became available in the U.S., only 63% of the population completed the primary series of an approved COVID-19 vaccine^4^. In contrast, Canada, Japan, and Italy reached vaccination rates of 70% and above by December 2021^5^. Despite growing evidence for the safety and effectiveness of vaccines, vaccine hesitancy remains influential and is driven by lack of trust in COVID-19 vaccines, concerns about side effects, and lack of trust in government^6^. Better understanding the factors that are associated with low vaccination uptake is crucial to address this problem.

Vaccine hesitancy is thought to be the primary driver of low COVID-19 vaccination rates in the U.S. MacDonald's 3C model describes vaccine hesitancy as influenced by confidence (in vaccine safety), complacency (low perceived risk of the disease), and convenience (vaccine availability and accessibility)^7^. To-date, survey-based studies have been the predominant method for examining COVID-19 vaccine hesitancy. Aw et al. summarized 97 of these survey-based studies in high-income countries and regions (39 of the articles were specific to the U.S.) and found that factors associated with higher COVID-19 vaccine hesitancy included younger age, females, non-white ethnicity, lower education, lack of recent history of influenza vaccination, lower self-perceived risk of contracting COVID-19, lesser fear of COVID-19, residing in rural areas, political inclination towards non-democrats, and not having chronic medical conditions^8^. These studies are valuable sources of individual level data and can explore psychological factors that impact hesitancy, but they are limited by relatively low sample size and sampling bias. Therefore, population level studies are needed to determine whether these survey findings are generalizable.

Most existing studies at the population level for the U.S. are at the county level and use linear models to examine the relationship between vaccine coverage and demographic features, like income, race, and political affiliation. Multiple studies found political affiliation to be a strong predictor, defined based on 2020 presidential election voting^9,10^. Other demographic features found to be associated with vaccination coverage were socioeconomic status^9,11^, race^9,11^, education level^6,11^, insurance coverage^9,11^, age^8^, vehicle access^6,12^, living in rural areas^13^, and population density^13^. Only two population-level studies incorporated data on information consumption. One study found that more viewership of Fox News during January and February 2020 was associated with lower weekly vaccination uptake between May and June 2021, a relationship that held even when political affiliation was controlled for Ref.^14^. Another study found that the percentage of COVID-19 vaccine-related misinformation shared on Twitter (also known as X after July 2023), in addition to increased GOP vote, was negatively associated with vaccine uptake rates^15^.

To analyze COVID-19 vaccine uptake and its determinants, our study uses a population-level statistical analysis conducted at the U.S. county level. We define a new variable derived from vaccination data, specifically, the percent of a county population that did not receive any dose of a COVID-19 vaccine by December 15, 2021^6^, which we refer to as *unvaccinated percentage.* While we are not directly measuring vaccine hesitancy, we believe this work will still provide insight on factors correlated with vaccine hesitancy, as previous work has shown a strong correlation between vaccine uptake and vaccine hesitancy^6^. In this study, we have designed our response variable to be the best proxy of vaccine hesitancy based on available vaccination data. Our variable reflects real world vaccination behavior at the population level, avoiding sampling biases from survey vaccine hesitancy data, and our selected time cutoff for measuring vaccine uptake minimizes the impact of non-hesitancy related factors. We implement weighted generalized additive models (GAMs) to identify the relationships between potential determinants and this COVID-19 unvaccinated percentage variable, and we include a more comprehensive set of factors potentially influencing vaccine hesitancy than previous work. The choice of variables and the model are described in detail in the following section.

## Data and method

This COVID-19 vaccination study is based on the latest publicly available data at the county level in the U.S. and uses a weighted generalized additive model (GAM). The response variable is COVID-19 unvaccinated percentage, and the independent variables cover eight categories, including COVID-19 epidemiological data, demographic, socioeconomic, and land use data, prior non-COVID vaccination behavior data, political affiliation data, select cable TV channel viewership ratings, and a Twitter misinformation variable (Table 1). The determinants for this study were chosen based on the set of factors previously identified to be associated with vaccine uptake in the literature, as well as our own hypotheses about factors that could potentially influence vaccine uptake, e.g., COVID-19 burden in a county and prior non-COVID-19 childhood vaccination rates. Each variable is defined in detail below. The correlation matrix is shown in the Supplementary Material (Fig. S1).

**Table 1:** Summary statistics for the raw data of the response variable and determinants.

Variable	N	Mean	SD	Source
Response variable				
COVID-19 unvaccinated percentage	3143	45.28	13.03	^16^
Demographic data				
Percentage of Black people	3143	8.72	14.11	^17^
Percentage of Latinx people	3143	9.79	13.68	^17^
Postsecondary education percentage	3143	53.68	10.72	^18^
Median age	3141	41.43	5.42	^17^
Socioeconomic data				
Uninsured percentage	3141	9.64	5.11	^17^
Average number of vehicles per household	3141	1.98	0.24	^17^
Median household income	3140	55,707.45	13,388.63	^17^
Political affiliation data				
Republican presidential vote percentage	3115	64.96	16.14	^19^
Land use data				
Population	3143	105,456.34	335,760.39	^17^
Population in urban counties	1133	251,129.27	527,897.79	^17^
Population in rural counties	2010	23,343.19	23,994.75	^17^
COVID-19 epidemiology data				
Cumulative COVID-19 case rate	3143	16,572.79	3999.03	^20^
Non-COVID-19 vaccination behavior				
MMR vaccination coverage	3101	0.93	0.08	^21^
Information consumption data				
FNC viewership	3071	0.75	1.33	^22^
CNN viewership	3071	0.27	0.42	^22^
MSNBC viewership	3071	0.27	0.37	^22^
Local news viewership	3071	4.92	2.21	^22^
Twitter misinformation percentage	904	0.39	0.49	^23^

## Data

### Response variable

*COVID-19 unvaccinated percentage* (UP) is our chosen response variable. UP is calculated as the partial vaccination rate (PVR) subtracted from 100%, where the PVR is defined as the percentage of people in a county who had taken at least one dose of Pfizer (Comirnaty) or Moderna (Spikevax)^16,24^. As our goal is to deepen our understanding of vaccine hesitancy or vaccine refusal, we chose to define our variable as the percent of the population that did not receive any COVID-19 vaccine doses, rather than the percent fully vaccinated. Defining our variable as the percent fully vaccinated would complicate the interpretation of the variable as vaccine refusal, since it would exclude those that were willing to get the first dose but did not get the second before our time cutoff. The PVR data used to compute UP is sourced from Georgetown University’s U.S. COVID-19 Vaccination Tracking website, which primarily relies on CDC data, supplemented with vaccination data from local health departments where CDC data is incomplete^16^. Vaccination data is not available for 69 counties in Alaska, Nebraska, Georgia, and Virginia, so these counties were excluded from the analysis. Additionally, since the Johnson & Johnson (J&J) vaccine only requires one dose to be fully vaccinated, the PVR excludes individuals who got the J&J vaccine. However, only 3.3% of vaccinations administered were J&J as of December 15, 2021^25^, so the exclusion of J&J vaccinations should have minimal impact on our conclusions.

While our response variable measures lack of vaccine uptake, not hesitancy directly, we believe that this work will still provide insight on factors correlated with vaccine hesitancy. Previous work found that COVID-19 vaccine uptake is strongly correlated with vaccine hesitancy, as measured by survey data^6^. Further, vaccine uptake rates reflect real world vaccination behavior at the population level, in contrast to vaccine hesitancy surveys which are available for a subset of locations around the U.S. and suffer from sampling biases. In addition, we selected the time cutoff of December 15, 2021 in order to minimize the impact of non-hesitancy factors on uptake, such as vaccine eligibility and accessibility. Therefore, our response variable serves as a reasonable proxy for vaccine hesitancy, and we think it is the best choice possible based on the data that is currently available.

### Explanatory variables

#### Demographic and socioeconomic data

All demographic and socioeconomic variables are sourced from publicly available datasets at the county level, from the U.S. Census Bureau’s 2020 Decennial Census^17^ and U.S. Department of Agriculture (USDA) Economic Research Service^18^. The percentage of Black people and the percentage of Latinx people represent the self-identified proportion of those races in each county. Postsecondary education percentage is measured as the percentage of adults with educational attainment more advanced than completing high school. The uninsured percentage is the percentage of people who reported not having health insurance. Additional metrics include median age, average number of vehicles per household and the median household income.

#### Political affiliation data

The political affiliation variable, defined as the percentage of voters who chose Donald Trump as their presidential candidate during the 2020 presidential election, is sourced from the MIT Election Data and Science Lab^19^. Previous work has found this data to be associated with vaccine hesitancy^9,10^. Compared with other political indicators, such as other election results, voter registration data, or public opinion polls, the presidential election has the highest voter turnout and the most policy influence. We hereby adopt this metric as a proxy of the political affiliation. It is referred as “Republican presidential vote percentage (%)” in the study.

#### COVID-19 epidemiology

In efforts to explore whether a county that experienced more burden from COVID-19 may be more willing to adopt preventative measures such as vaccination, we incorporate a variable to capture a county's historical COVID-19 infection rate. Specifically, to measure historical COVID-19 burden, we use the cumulative number of COVID-19 cases per 100,000 people as of December 15, 2021 from the Johns Hopkins University Center for Systems Science and Engineering (CSSE) COVID-19 GitHub^20^. In order to remove outliers, values that were more than 4 standard deviations above the mean were excluded.

#### Non-COVID-19 vaccination behavior

Since MMR vaccination coverage is an indicator of vaccine acceptance before COVID-19, we hypothesize that higher (pre-pandemic) MMR vaccine uptake rates may be associated with higher COVID-19 vaccine coverage. Previous work has shown a strong association between MMR coverage and vaccine hesitancy^26,27^. In most U.S. states, the MMR vaccine is required for children to attend public school, making MMR coverage a strong indicator of anti-vaccination behavior. While this data reflects the results of parents making vaccination decisions for their children, in contrast to measuring COVID-19 uptake among mostly adult populations, the decision to forgo mandatory childhood vaccinations is indicative of strong hesitancy that we hypothesize may transfer to other vaccines. To test this hypothesis, we incorporate a variable in this analysis that is based on the MMR vaccination coverage rates of children in kindergarten in 2019, data that we gathered in a previous study^21^.

#### Information consumption

A set of variables intended to capture the potential role of information consumption on vaccine choice includes four television viewership rating variables and a Twitter misinformation variable. The county level viewership ratings (RTG) % for four major channels, namely FNC (Fox News Channel), CNN (Cable News Network), MSNBC (Microsoft National Broadcasting Company), and local news, are sourced from Nielsen Media, where RTG is measured by the estimated percentage of households tuned to a specific viewing source, e.g., news channel. The four viewership variables were computed as the average of the monthly viewership ratings for each channel from February to November 2021. January 2021 data were excluded due to anomalies caused by the January 6th U.S. Capital Attack. Nielsen data is not available for several counties in Virginia and Alaska and all counties in Hawai’i, so these 72 counties are excluded from the analysis. The analysis also excludes outliers, defined as those counties with cable viewership values that are more than 4 standard deviations away from the mean. Additionally, within the model each of the cable TV viewership variables was standardized to a mean of 0 and standard deviation of 1, to provide a more interpretable understanding of the relative position of each county’s rating.

Another information consumption variable included in the model is the Twitter misinformation variable. This variable is intended to capture the prevalence of COVID-19 vaccine misinformation in circulation on Twitter during a time that likely influenced behavior during the study period. The variable is based on a previous study by Pierri et al., who provided a variable that is representative of the percent of COVID-19 vaccine-related tweets that contain links to low credibility sources at the county level^15,23^. This variable has some limitations, as it is based on only the set of Twitter accounts that can be geolocated. To ensure a large enough sample size for a reliable estimate, counties with less than 50 geolocated accounts are not included, which results in a data set that includes 904 counties. After excluding outliers, defined as values that are more than 4 standard deviations from the mean, we have 855 counties. An analogous data set is also available with a minimum of 10 and 100 geolocated accounts, but we opted to use the cutoff of 50 to balance having a more representative sample size of accounts per county with the number of counties we can include in our analysis. Due to the limited number of counties that this data is available for, a separate sub-analysis is conducted that includes this variable (Fig. 2).

#### Land use data

Various land-use variables are sourced from the U.S. Census Bureau, namely the population size and the number of residents in rural or urban areas for each county^28^. These variables are used to cluster counties for the sub-analyses, which are further described in the methods section. For the cluster-based analysis we categorize counties into mutually exclusive sets based on (1) population quartiles and (2) a binary rural or urban classification. For the binary classification, a county is classified as rural if the majority of the population is designated to live in areas classified as rural and otherwise classified as urban.

### Statistical models

We use a Generalized Additive Model (GAM) to explore the relationship between each county's unvaccinated percentage and the aforementioned variables. GAMs provide a balance between model complexity and interpretability, and critically, they can reflect the relative importance of different features^29,30^. Specifically, GAMs model the response variable as the sum of unknown smooth functions of covariates, and unlike Generalized Linear Models (GLMs), GAMs offer the capability to model nonlinear relationships between variables. For example, a linear regression model may show an overall positive correlation between an input variable and the response variable, but using a GAM on the same data may reveal a more nuanced relationship, like a strong positive trend in some ranges of the input variable and a weak negative trend elsewhere. Due to the complex nature of relationships between our variables and vaccination uptake, GAMs are a more appropriate choice than linear models, since they can capture both linear and nonlinear relationships.

### Primary model

The proposed GAM is fitted to the unvaccinated percentage as the response variable, which is assumed to have a Gaussian distribution, and a log link. REML (restricted maximum likelihood) is used to estimate smoothing parameters, which returns relatively reliable and stable results. Specifically, the model in our primary analysis has the following form:

$${Y}_{i}\sim Gaussian(\mu )$$

$${\text{log}}\left(\mu \right)\sim {s}_{1}\left(cumulative \,COVID-19\, case\, rate\right)+ {s}_{2}\left(percentage\, of \,Black\, people\right)+ {s}_{3}\left(percentage\, of \,Hispanic\, people\right)+ {s}_{4}\left(postsecondary\, education\, percentage\right)+ {s}_{5}(median \,household \,income) + {s}_{6}(median \,age) + {s}_{7}(vehicles\, per\, household) + {s}_{8}(uninsured\, percentage) + {s}_{9}(MMR\, coverage) + {s}_{10}(std(FNC\, viewership) + {s}_{11}(std(CNN \,viewership)) + {s}_{12}(std(MSNBC\, viewership) + {s}_{13}(std(Local\, News\, viewership) + {s}_{14}(Republican \,presidential \,vote \,percentage)$$

where *Y*_*i*_ denotes the unvaccinated percentage for each county *i*. The model is a sum of smooth functions ${s}_{i}$, and each smooth function consists of a number of basis functions (*k*). Sensitivity analysis that varies the number of basis functions was conducted. A value of *k* = 3 for each smooth function was found to provide the optimal balance between preventing both underfitting and overfitting of the model and maximizing interpretability of the results. Additionally, the GAM model is weighted to prevent the highly imbalanced county population distribution from skewing the results. The weight is computed by normalizing each county’s population by the average county population, taking a log transformation to adjust for the skewness. The weight implemented in the primary analysis is defined as:

$$weigh{t}_{i}=\frac{{\text{log}}(po{p}_{i})}{mean({\sum }_{i}{\text{log}}(po{p}_{i}))},$$

where *i* is the county index. The primary model is run for 2885 counties (reduced from the full set of counties due to missing data and data quality issues referenced previously).

As noted previously, the Twitter misinformation variable, ${s}_{15}(Twitter \,misinformation)$, is only available for 855 counties, and is therefore run as a separate model using the same general function and weights as the primary model, but with the additional determinant included.

### Sensitivity analysis

In addition to the primary model presented above, we conduct sub-analyses to determine how the relationship between unvaccinated percentage and its associated factors varies across urban versus rural counties. In the U.S., vaccination uptake was substantially lower in rural areas^31^. Multiple studies have examined the reasons for this discrepancy. Some factors associated with lower vaccination in rural areas were captured in our study, including lower educational attainment, voting for Trump in the 2020 election, and lower insurance coverage^32^. However, other relevant factors could not be incorporated in our study, including that rural residents have a lower perceived risk of COVID-19, higher vaccine hesitancy, and are less likely to adopt covid risk mitigating behaviors^32,33^. In order to better understand the influence of different factors in rural versus urban counties, we complete two cluster-based sub-analyses: one clustered by rural versus urban counties and another clustered into four quartiles based on population size, as described below. Due to the difficulty of neatly separating counties into urban or rural, we provide the population size cluster analysis to confirm that our urban–rural analysis is accurately capturing differences between urban and rural counites, which broadly aligns with higher versus lower county populations.

1. Land-use cluster-based analysis: Counties are clustered into two groups based on their primary land use pattern, namely as urban or rural counties. Two independent weighted GAM models are run, one for each group. The rural model includes 1,835 counties, and the urban model includes 1,050 counties.
2. Population cluster-based analysis: Counties are grouped into quantiles based on their population size. Four independent GAM models are generated, one for each distinct quantile. The respective models contain 664, 721, 739, and 761 counties ranging from the smallest to largest population size groups. GAMs are implemented without weights for each group in this sub-analysis, because the weighting is based on population size.

We evaluate the goodness-of-fit by conducting a diagnostic analysis for each model and sub-model. These evaluations include the Q-Q plots, histograms of residuals, mapping of residual values versus predicted values, and mapping of response against fitted values. The diagnostic analysis outcomes for the primary model are presented in the Supplementary Material (Fig. S2). The concurvity in the primary model is also measured to ensure pairwise values remain below 0.8 and avoid cases in which one variable is a smooth function of another. The outcomes of the diagnostic analysis demonstrated consistency in fit and performance across all models.

## Results

**Figure 1:**
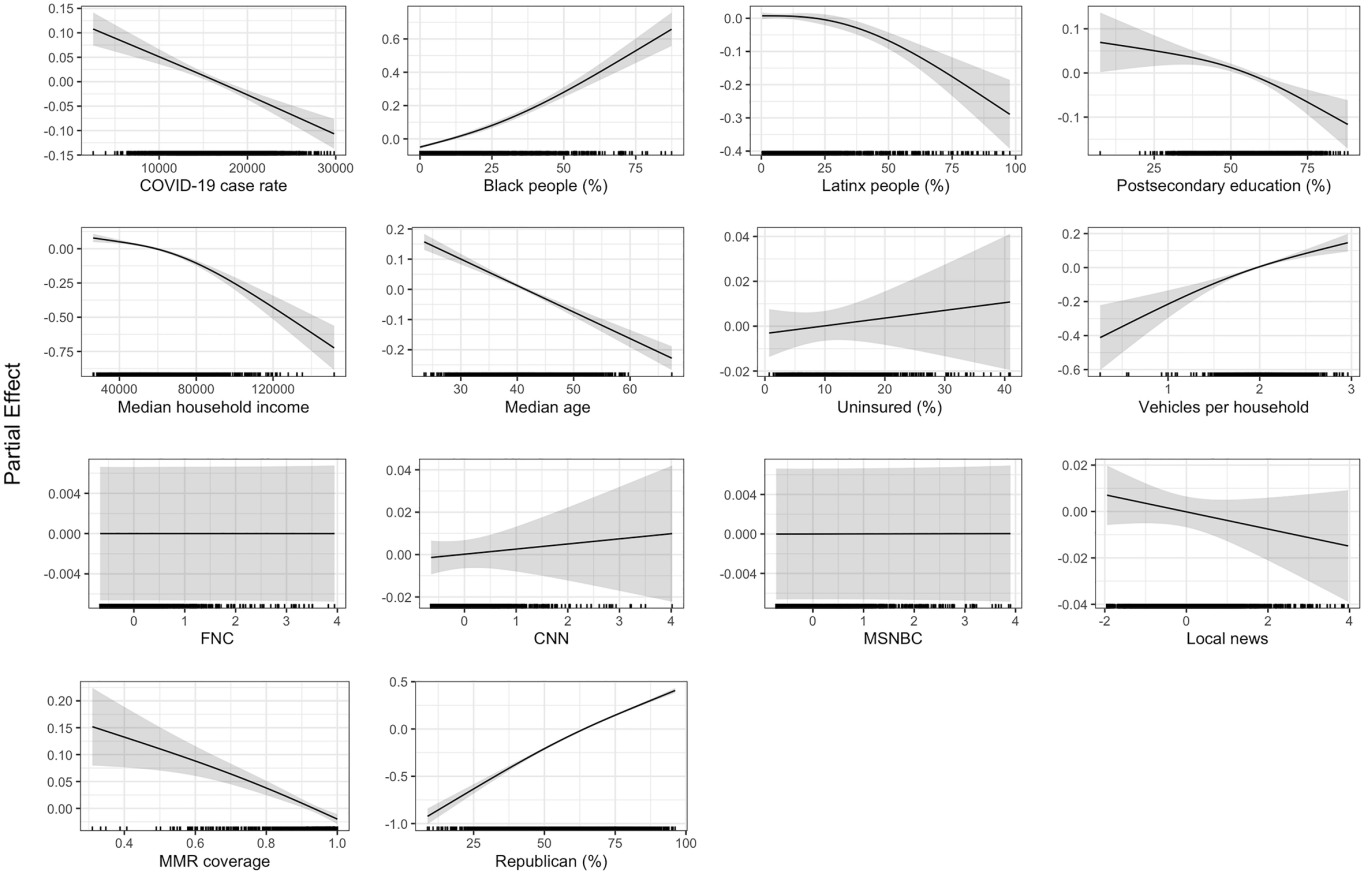
GAM results for the primary model. The shaded regions in each curve indicate the 95% confidence intervals, and the points at the bottom of each subplot indicate the distribution of each determinant.

**Figure 2:**
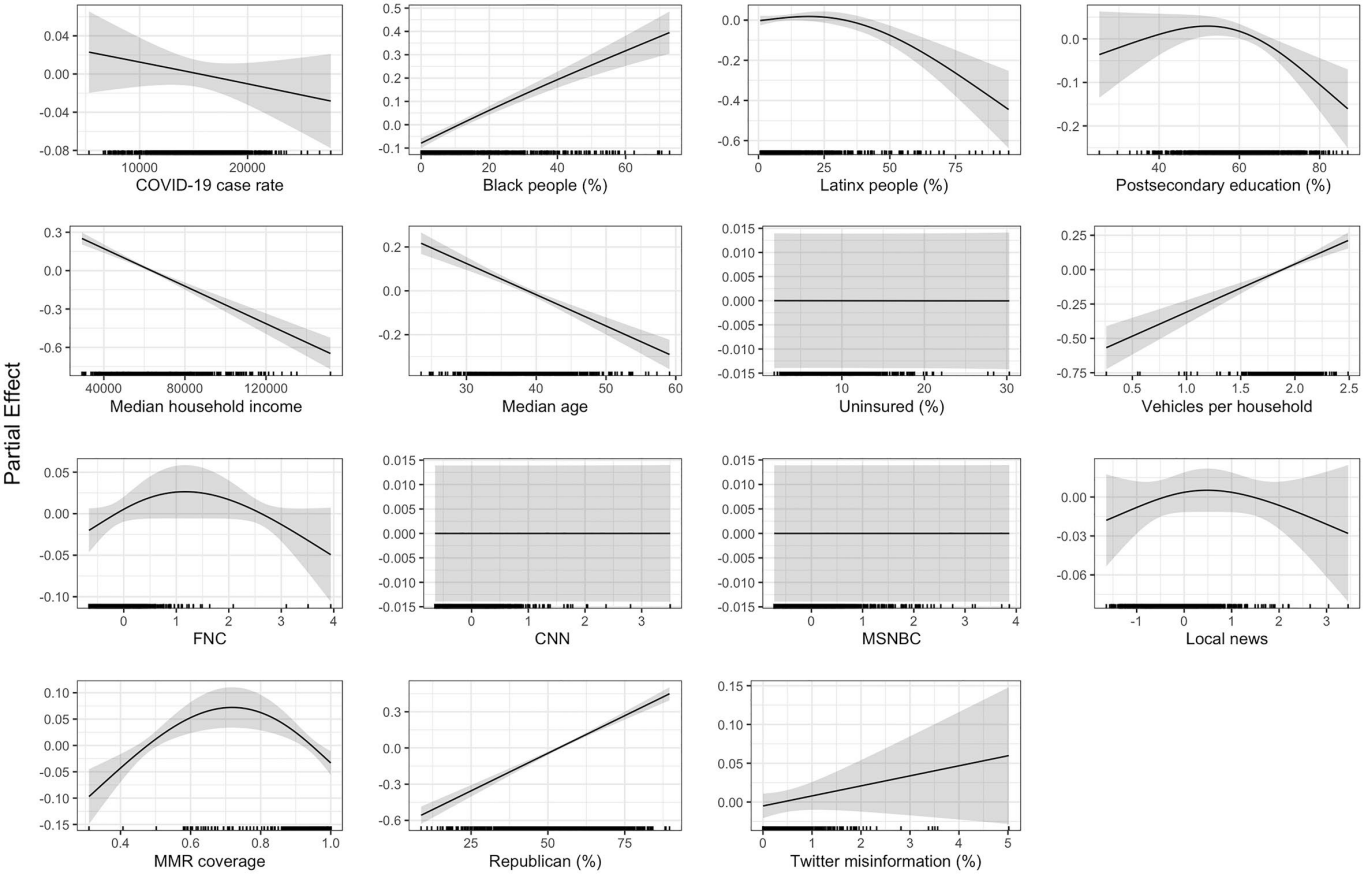
Results of the generalized additive models with Twitter misinformation (%) with 855 counties in the model. The shaded regions in each curve refer to the 95% confidence intervals, and the points at the bottom of each subplot indicate the distribution of each determinant.

**Figure 3:**
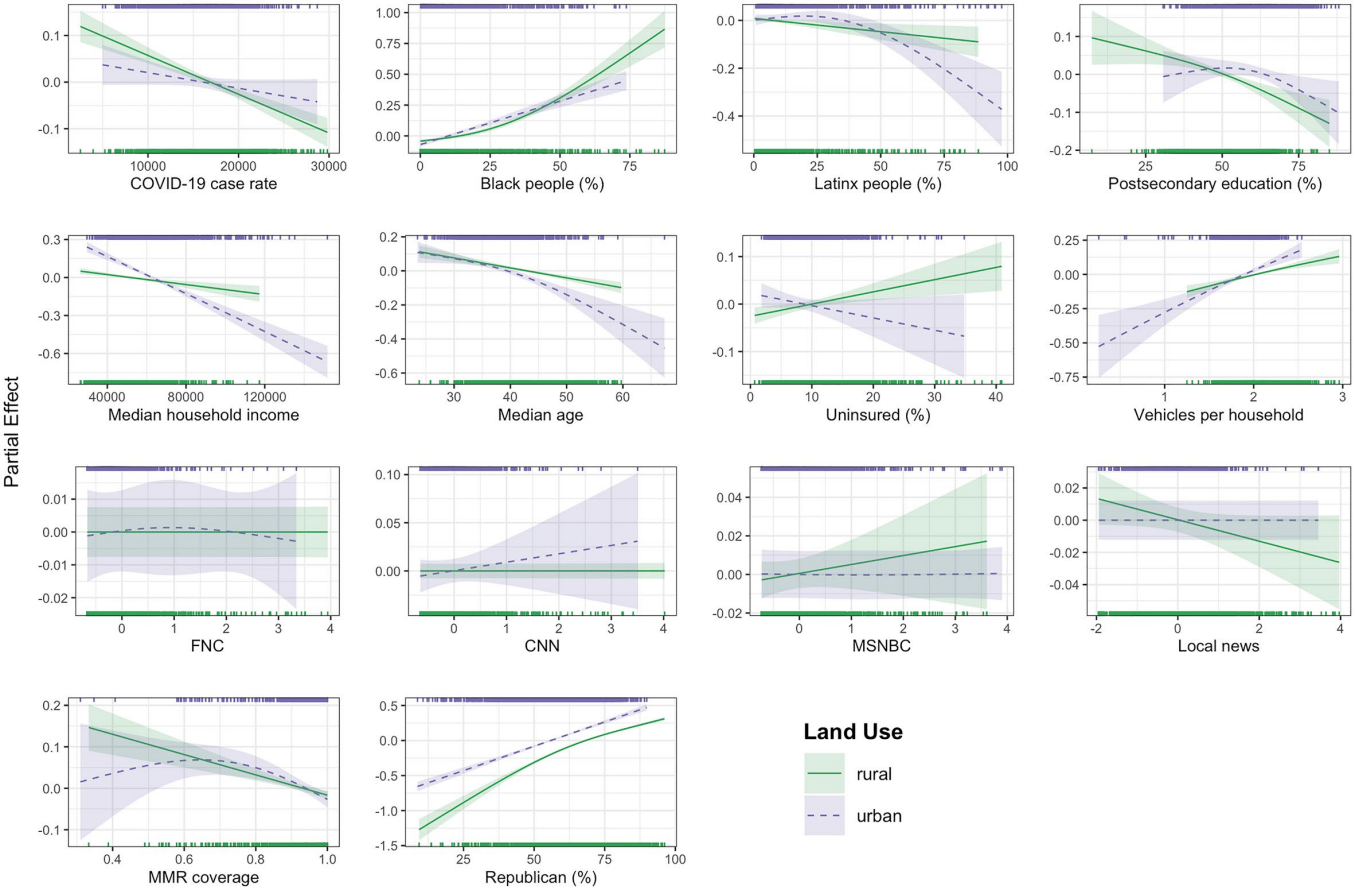
Results of the generalized additive models clustered by land use (i.e., rural counties vs. urban counties). The shaded regions in each curve indicate the 95% confidence intervals, and the points at the top and bottom of each subplot indicate the distribution of each determinant by cluster.

The GAM results are presented for each analysis: (1) the primary model for 2885 counties in the U.S. (Fig. 1), (2) the sub-model that includes a Twitter misinformation variable for 855 counties (Fig. 2), (3) the land-use cluster-based analysis with separate models for counties classified as urban or rural (Fig. 3), and (4) the population cluster-based analysis with separate models for counties grouped based on population size in the Supplementary Material (Fig. S3).

### Primary model of COVID-19 vaccination rates and associated factors in the U.S.

Figure 1 contains the GAM results for the primary model that includes the majority of U.S. counties. The factor with the strongest positive association with COVID-19 unvaccinated percentage in our model is Republican presidential vote (%), i.e., the percentage of voters choosing Donald Trump as their presidential candidate during the 2020 presidential election. Two other determinants positively associated with unvaccinated percentage are the percentage of Black people and the average number of vehicles per household; however these associations are not as strong as the Republican presidential vote (%). Multiple variables are found to be negatively associated with unvaccinated percentage, namely cumulative COVID-19 case rate, percentage of Latinx people, postsecondary education percentage, median household income, median age, and MMR vaccination coverage, although the strength of the associations varies, with the strongest negative association appearing for median household income. In contrast, the uninsured percentage and the cable TV viewership variables do not show a statistically significant relationship (*p* < 0.05) with unvaccinated percentage in the primary model (see Table S1 in the Supplementary Material). Results with a unified y-axis range are shown in the Supplementary Material (Fig. S4), which further illustrates the strong relative association of Republican presidential vote percentage compared to other determinants.

### Sub-model incorporating Twitter misinformation rates

Figure 2 presents results from the sub-model for 855 counties that includes the Twitter misinformation variable from Pierri et al.^15^, which represents the proportion of COVID-19 vaccine-related tweets sharing low credibility sources in a county. In contrast to previous work^15^, our study does not find a significant association between the Twitter variable and unvaccinated percentage. The results from both the primary model and this sub-analysis are broadly consistent, with the only differences being that for the model with Twitter misinformation, the cumulative COVID-19 case rate is not significant, Fox News viewership has a significant inverted U-shaped trend, and the postsecondary education percentage has a slight inverted U-shaped trend. The trends for COVID-19 case rate and postsecondary education are consistent with our findings in urban counties and counties with larger populations (see cluster-based analysis), which reflects that the sample of counties that have Twitter misinformation data are biased towards larger urban counties. The inverted shape of the Fox News trend is driven by a very small number of counties with viewership values greater than 2, while the positive slope is consistent with the vast majority of counties included in the model.

### Land-use cluster-based sensitivity analysis

Figure 3 shows the results of the land use cluster-based sensitivity analysis, which fits separate models for rural and urban counties to determine if the relationships between the variables and unvaccinated percentage vary across urban versus rural counties. The sub-models reveal the same significant variables as the primary model with a few exceptions. For rural counties there is a positive association between the uninsured percentage and unvaccinated percentage, compared to no significant relationship in the primary model and for urban counties. The rural counties also have a negative association between local news viewership and unvaccinated percentage, while the primary and urban counties have no significant relationship between these variables. When comparing the urban and rural counties, rural counties have a stronger negative association between cumulative COVID-19 case rate and unvaccinated percentage, while urban counties show stronger trends for the negative association between percentage of Latinx people and unvaccinated percentage, and the negative association between median household income and unvaccinated percentage. The trends for postsecondary education and MMR vaccination coverage are more complex in urban counties, forming a more uncertain, slightly inverted U-shaped trend, compared to a more linear trend for rural counties. The slightly positive trend for counties with MMR coverage less than 60% is driven by only three counties, and as evidenced by the wide uncertainty interval, is not a significant trend.

### Population cluster-based sensitivity analysis

Results for the population cluster-based sensitivity analysis is presented in the Supplementary Material (Fig. S3). Four independent models are fit for the sets of counties clustered by population quartile to further assess the robustness of the associations identified in the land-use sub-analysis. The results from the population sub-models are consistent with the models for urban and rural counties. Specifically, the negative association between cumulative COVID-19 case rate and unvaccinated percentage is higher in counties with smaller population, the percentage of Latinx people’s negative association is stronger in counties with larger population, local news viewership only has a significant negative association in smaller counties, and postsecondary education percentage has a stronger negative association in smaller counties. For MMR coverage, the smallest quartile shows no relationship with unvaccinated percentage, the middle two quartiles have a clear negative association, and the largest quartile has an inverted U-shaped trend. Like the urban counties model, the positive association in the largest quartile when MMR coverage is less than 60% is driven by a very small number of counties with large population size and low MMR vaccination coverage.

## Discussion

Across all models presented, political affiliation, namely the percentage of voters who voted for Donald Trump in the 2020 presidential election, has the strongest association with COVID-19 unvaccinated percentage in the U.S. This result is consistent with previous studies at the individual level^34,35^ and at the population level^9,10,15^.

For demographic variables, a high percentage of Black people is positively associated with unvaccinated percentage, while a high percentage of Latinx people is negatively associated with unvaccinated percentage. These results are consistent with previous studies^9,11,15^. While the reasons for these group differences in vaccine uptake are difficult to pinpoint, Frisco et al. shed light on this question by analyzing survey data. They find that vaccine hesitancy is higher among Black populations than white populations, likely due to the legacy of racism Black Americans have faced in medicine and medical research. For Latinx populations, they found levels of vaccine hesitancy to be about the same as white populations. However, Latinx populations experienced higher burden from COVID-19, which translated into more vaccine acceptance. While Black populations also experienced high burden from COVID-19, this influence did not overcome baseline vaccine hesitancy^36^.

Other demographic and socioeconomic factors negatively associated with unvaccinated percentage include median age, median household income, and postsecondary education percentage, while the average number of vehicles per household has a positive association. These results are also consistent with previous work. Older people were able to access the vaccine earlier and are more susceptible to COVID-19, which likely increased their vaccination uptake^8^. Higher levels of educational attainment^6,11^ and higher income^9,11^ were associated with increased vaccination uptake. While previous work found that greater insurance coverage is broadly associated with higher vaccine uptake coverage^9,11^, our results identified this relationship only in rural counties. This may reflect that health insurance was more influential for certain subpopulations with more limited access to vaccines and healthcare in general, as is the case in rural counties^37^. More vehicles per household was associated with decreased vaccination uptake, which is consistent with previous work that shows higher vaccine coverage to be associated with lower percentages of households with a vehicle^6,11^. This finding seems counterintuitive since vehicle access suggests easier access to vaccines. However, urban counties have less vehicles on average than rural counties, but urban residents typically enjoy closer proximity to vaccine centers and transit options other than private vehicles. Therefore, this relationship may be capturing confounding factors such as urban residents typically having easier access to vaccines and less vaccine hesitancy.

Our study found that higher COVID-19 case rates are associated with lower unvaccinated percentage. We hypothesize that in counties that experienced a higher historical case burden of COVID-19, individuals were more aware of the risks of COVID-19 and thus more willing to seek out preventative measures like vaccination. However, this relationship was weaker in urban counties and was insignificant in our model with a Twitter misinformation variable, which is biased towards counties with larger populations. In most cases, historical burden of COVID-19 appears to influence the perceived risk versus the reward of vaccination and encourage uptake, except in counties with larger populations, in which other determinants were more important.

Results from our analysis revealed a negative association between prior non-COVID-19 vaccination behavior, measured by MMR vaccination coverage, and COVID-19 unvaccinated percentage. This relationship was stronger in rural counties than urban counties. The MMR vaccination coverage is an indicator of vaccine acceptance before COVID-19, which is based on vaccination coverage of children in kindergarten, and reflects their parents’ acceptance of recommended childhood vaccinations. That higher MMR vaccination coverage was associated with higher COVID-19 vaccine uptake suggests that vaccine hesitancy in the U.S. was not necessarily specific to COVID-19 vaccines, and that the same populations that were historically hesitant towards recommended childhood vaccinations (for their children) were also hesitant towards COVID-19 vaccination (for themselves). These results suggest that successful vaccination campaigns can help increase vaccination uptake more broadly, but they must address the more complex issue of general hesitancy towards vaccines, rather than just concerns around a specific disease. In order to better understand vaccination behavior, we need high quality, publicly available vaccination data at high resolutions for non-COVID-19 diseases.

Previous work found that cable news viewership had a significant relationship with vaccine uptake. However, results from our more comprehensive model, which includes a broader scope of variables that impact vaccine uptake, did not indicate consistent and significant relationships for these cable news viewership variables. The only significant relationships with unvaccinated percentage found were for local news viewership (negative association) in rural counties and Fox News viewership (uncertain, inverted U-shaped trend) in the sub-model with a Twitter misinformation variable. In contrast, a previous study on viewership of cable TV found evidence of causality between Fox News viewership and lower weekly vaccine uptake between May and June 2021, a relationship that holds when controlling for self-reported political affiliation^14^. In addition to considering a more limited range of variables, the study focuses on a smaller timespan and defines political affiliation using the Gallup Polling Series 2012–2019, rather than 2020 presidential election voting.

Our study also found no significant relationship between the Twitter misinformation variable provided by Pierri et al. and COVID-19 unvaccinated percentage, which also contrasts previous findings^15^. Pierri et al. found that this Twitter misinformation variable was the most significant predictor for vaccine coverage at the state level, followed by political affiliation^15^, however, at the county level political affiliation was shown to be a stronger predictor of vaccine coverage than Twitter misinformation. The differences in our findings can be attributed to the following factors: the timepoints of vaccination data (March 2021 versus December 2021 in our study), a different number of counties included in the analysis (548 versus 855 in our study), differences in the modeling approaches employed (linear regression versus GAMs in our study), and our inclusion of a broader array of variables. It's important to note, however, that our findings do not necessarily indicate that information consumption does not impact vaccine uptake. Instead, it is more likely that we do not yet have data that accurately captures information consumption patterns that influence health-related behaviors. Future work should focus on obtaining data that covers a more representative sample of information shared online, like increasing the number of tweets that can be geolocated or capturing other social media platforms, and employing natural language processing techniques in order to get a deeper understanding of the types of misinformation shared, as in Broniatowski et al.’s topic modeling analysis of COVID-19 vaccine information shared on Facebook^38^.

There are several limitations of this study. First, we defined our response variable as the percent of the population that did not receive any vaccine doses, which reflects the absence of vaccine uptake, not necessarily people’s attitudes towards vaccines. However, we believe our work still provides insights on the potential drivers of vaccine hesitancy. Previous work has shown a strong correlation between vaccine uptake and vaccine hesitancy^6^, and our response variable reflects real world vaccination behavior at the population level, in contrast to vaccine hesitancy survey data, which are available for a subset of locations around the U.S. and suffer from sampling biases. In addition, vaccination uptake is affected by multiple non-hesitancy factors, including vaccine accessibility, eligibility, personal risk of adverse vaccine effects, and vaccine mandates from local health departments, schools, or workplaces. To minimize the effect of these other factors, we calculate our response variable based on vaccination uptake as of December 15, 2021. This time cutoff is eight months after the vaccine became available to all adults in the U.S., at which time the impact of eligibility and accessibility-related factors would be minimal, and thus vaccine hesitancy would be the main driver of vaccine refusal. Second, attitudes toward vaccines evolved over time due to both scientific and anecdotal influences. Our study considers data up until December 2021 and does not account for variations over time. Third, our findings are only applicable to the U.S. Fourth, our analysis is conducted at the population level, therefore our results do not reflect any individual level findings. Fifth, GAMs are vulnerable to overfitting, especially to outliers, as shown in the MMR plot for urban counties and the Fox News plot in the model with Twitter misinformation. To counteract this tendency, we have removed outliers for variables when necessary and explicitly discussed when trends are not significant due to these issues. Finally, our analysis is driven by statistical correlations and therefore cannot make any claims about causal relationships.

## Conclusion

This study examines 3,000 U.S. counties covering over 300 million people to analyze the determinants associated with COVID-19 vaccine uptake in the U.S. In spite of the inclusion of multiple variables that would intuitively influence vaccination decision-making, such as historical COVID-19 burden, non-COVID-19 vaccination uptake, and a variable on the prevalence of COVID-19 vaccine misinformation on Twitter, we found political affiliation, defined by voting rates for Donald Trump in the 2020 election, to be the most strongly associated variable with vaccine uptake. These findings highlight the harms of the politicization of COVID-19 in the U.S. and its influence on public health decision-making, most critically, during the time period when vaccination was the most powerful tool for fighting COVID-19 and the burden from COVID-19 was at a peak. Future efforts to reduce vaccine hesitancy must confront the influence of political polarization on vaccine attitudes. In addition, our results identified the second strongest determinant of vaccine uptake, behind political affiliation, to be median income. We also found significant associations with race, education level, and vehicle access. Our study adds more evidence to the multitude of research showing that socioeconomic and demographic factors are consistently associated with public health outcomes of interest, which underscores the longstanding role of inequality in the U.S. driving disparate health outcomes. To combat vaccine hesitancy, we must address the underlying health inequities in society, including increasing access to quality healthcare and working to rebuild trust in the medical system.

Further interventions to improve COVID-19 vaccine uptake at the population level are difficult to recommend. Some reviews have summarized efforts to increase COVID-19 vaccination intent and uptake in the U.S. and other countries, such as educational interventions, incentives, policies, communication strategies, and increasing access^39,40^. In spite of the plethora of work addressing vaccine hesitancy interventions, reviews conducted both before and after COVID-19 have concluded that we have limited evidence on what kinds of interventions are effective, due to the difficulties of directly measuring the impact of these interventions and that the most effective interventions are tailored to specific populations, which complicates the ability to make generalizable claims of effectiveness^39–44^. It is especially concerning that there is a lack of research showing effective interventions for the current information ecosystem, in which social media has fundamentally changed the way information spreads, public health has been politicized, and discourse on any politics-adjacent topic has become polarized. What we do know is that personalized interventions for different populations are critical, and that we need to intervene at multiple levels of the information ecosystem: at the level of pieces of information, the individual level, the communication and community engagement level, and the institutional and structural level^45^. In conclusion, our study contributes to better understanding the drivers of vaccine hesitancy in order to inform future efforts to increase vaccination uptake, but there are critical gaps in the literature on how to address vaccine hesitancy.

### Supplementary Information

Supplementary Information.

## Data Availability

The data utilized in the study, along with the corresponding code, have been published on GitHub: https://github.com/CSSEGISandData/covid19_vaccine_hesitancy_study. However, in compliance with the redistribution rules of Nielsen, the cable TV viewership data was excluded from the shared dataset.
