# Supplementary material for: A population level study on the determinants of COVID-19 vaccination rates at the U.S. county level
Source: Sci Rep. 2024 Feb 21;14:4277. doi: 10.1038/s41598-024-54441-x (PMC10881504; doi:10.1038/s41598-024-54441-x)
Supplement: Supplementary file 1 — Supplementary Information. [file 41598_2024_54441_MOESM1_ESM.docx]

Supplementary material for

**A Population Level Study on the Determinants of COVID-19 Vaccination Rates at the U.S. County Level**

Ensheng Dong^1,2,*^, Kristen Nixon^1,2^, Lauren M. Gardner^1,2,3^

^1^Department of Civil and Systems Engineering, Johns Hopkins University, Baltimore, MD 21218, USA.

^2^Center for Systems Science and Engineering, Johns Hopkins University, Baltimore, MD 21218, USA.

^3^Department of Epidemiology, Johns Hopkins Bloomberg School of Public Health, Baltimore, MD, 21205, USA.

* Email: [edong1@jhu.edu](mailto:edong1@jhu.edu)

**Table of Contents**

**Figure S1.** Correlation matrix among the outcome and the determinants.

**Figure S2.** Model evaluations for the GAM result of the primary model.

**Figure S3.** Results of the generalized additive models with clusters. Counties clustered by population size, with Q1 the smallest and Q4 the largest.

**Figure S4.** GAM results for the primary model with unified y-axis range.

**Table S1.** P-values for significance of smooth terms in each model. Values less than 0.05 are marked with an asterisk (*).


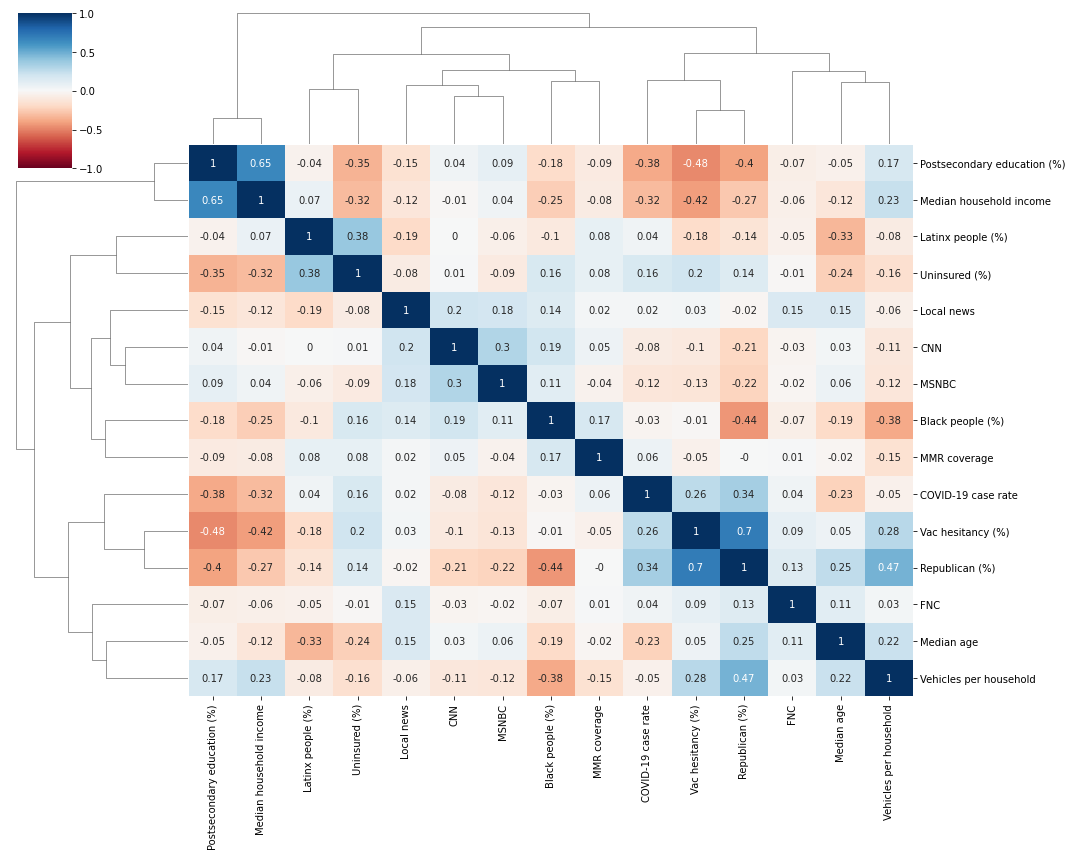


**Figure S1.** Correlation matrix among the outcome and the determinants for data in the primary model.


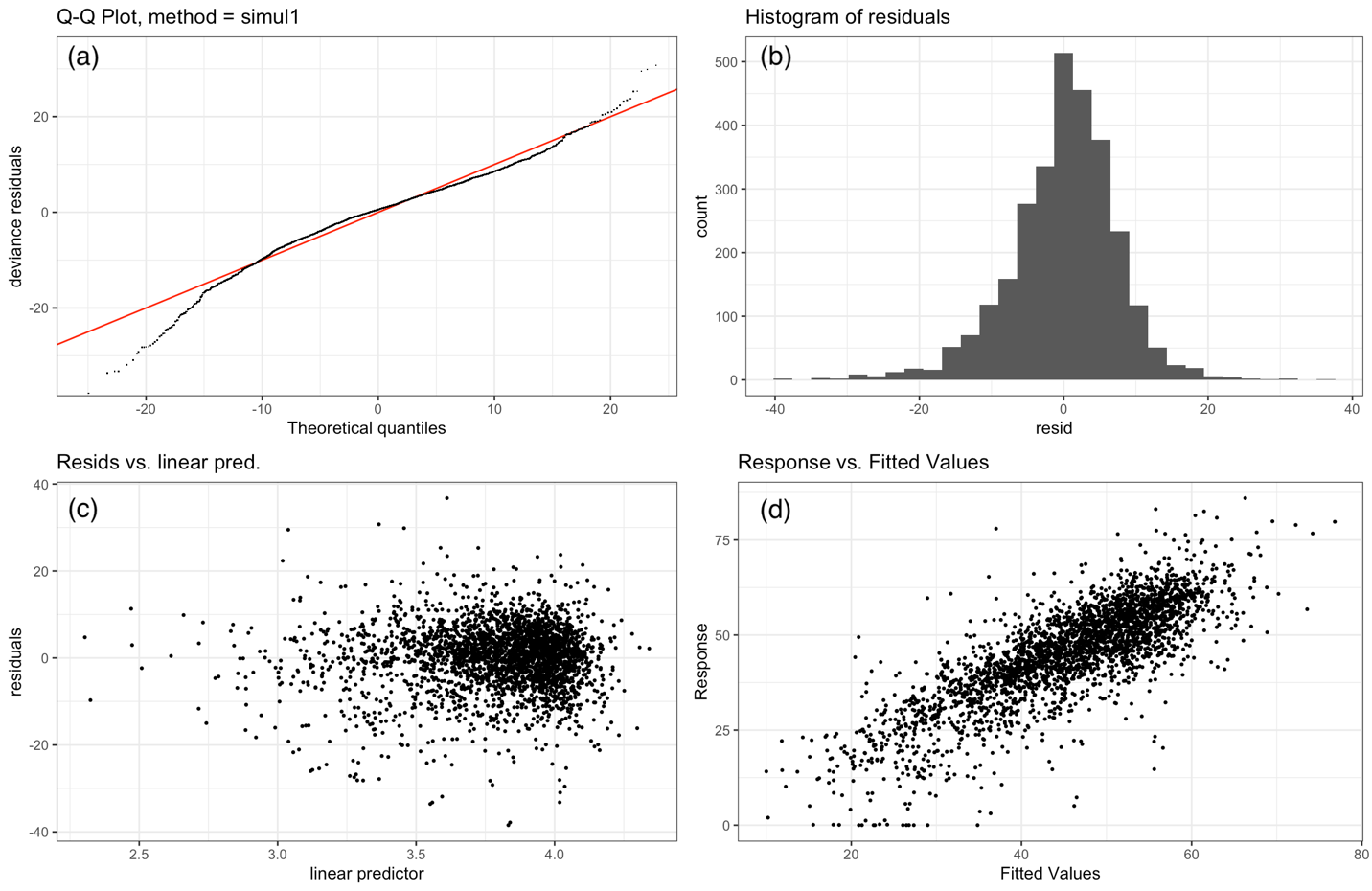


**Figure S2.** Model evaluations for the GAM result of the primary model. (a) Q-Q plot; (b) histogram of residuals; (c) plot of residual values versus predicted values; (d) plot of response against fitted values.


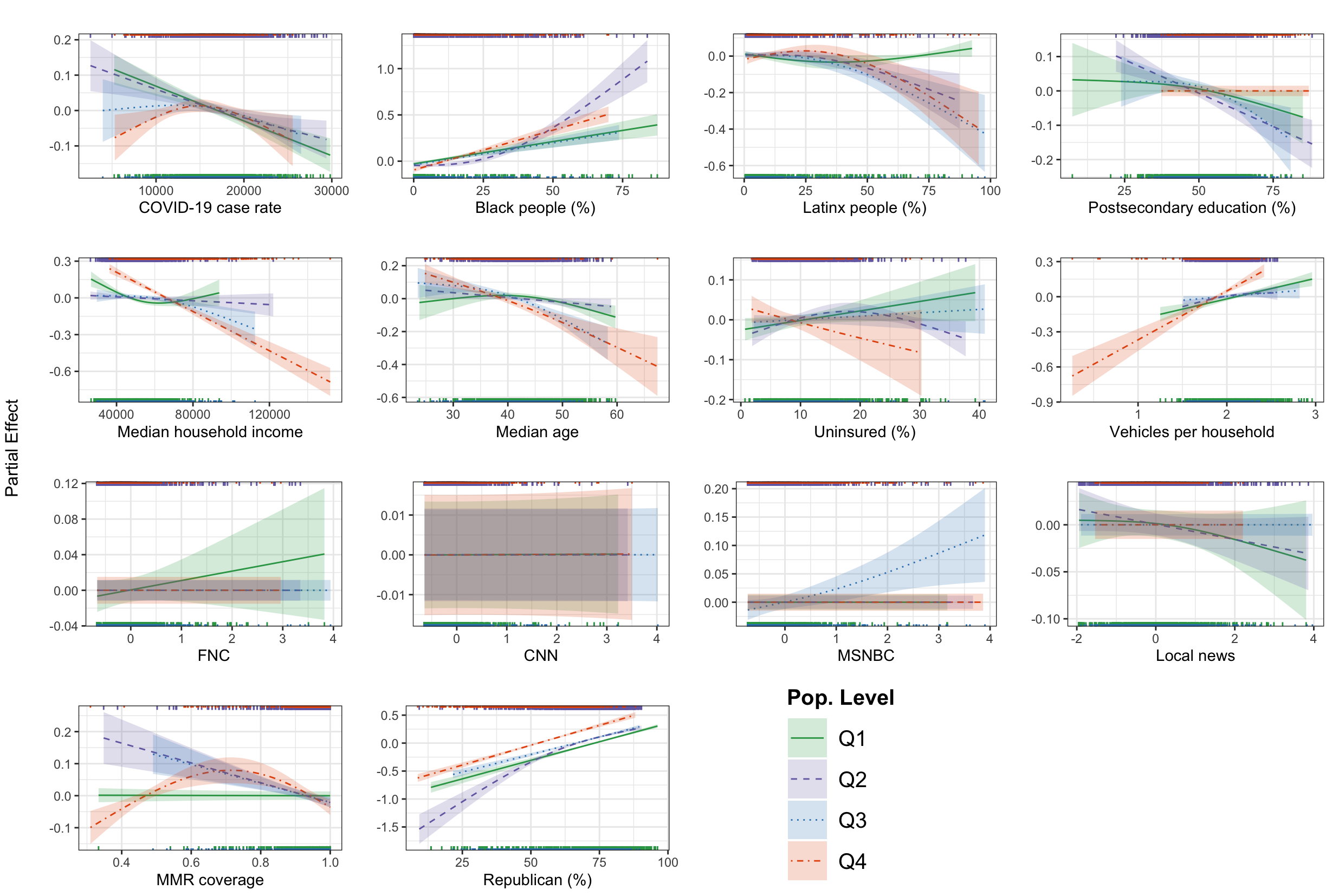


**Figure S3.** Results for the population cluster-based sensitivity analysis. Counties are clustered into quartiles based on population size, with thresholds at 10,830 (similar size to Sussex, VA), 26,000 (similar size to Staunton, VA), and 68,000 (similar size to Madison, NY). Q1 is the smallest quartile, and Q4 the largest. The shaded regions in each curve refer to the 95% confidence intervals, and the points at the top and bottom of each subplot indicate the distribution of each determinant.


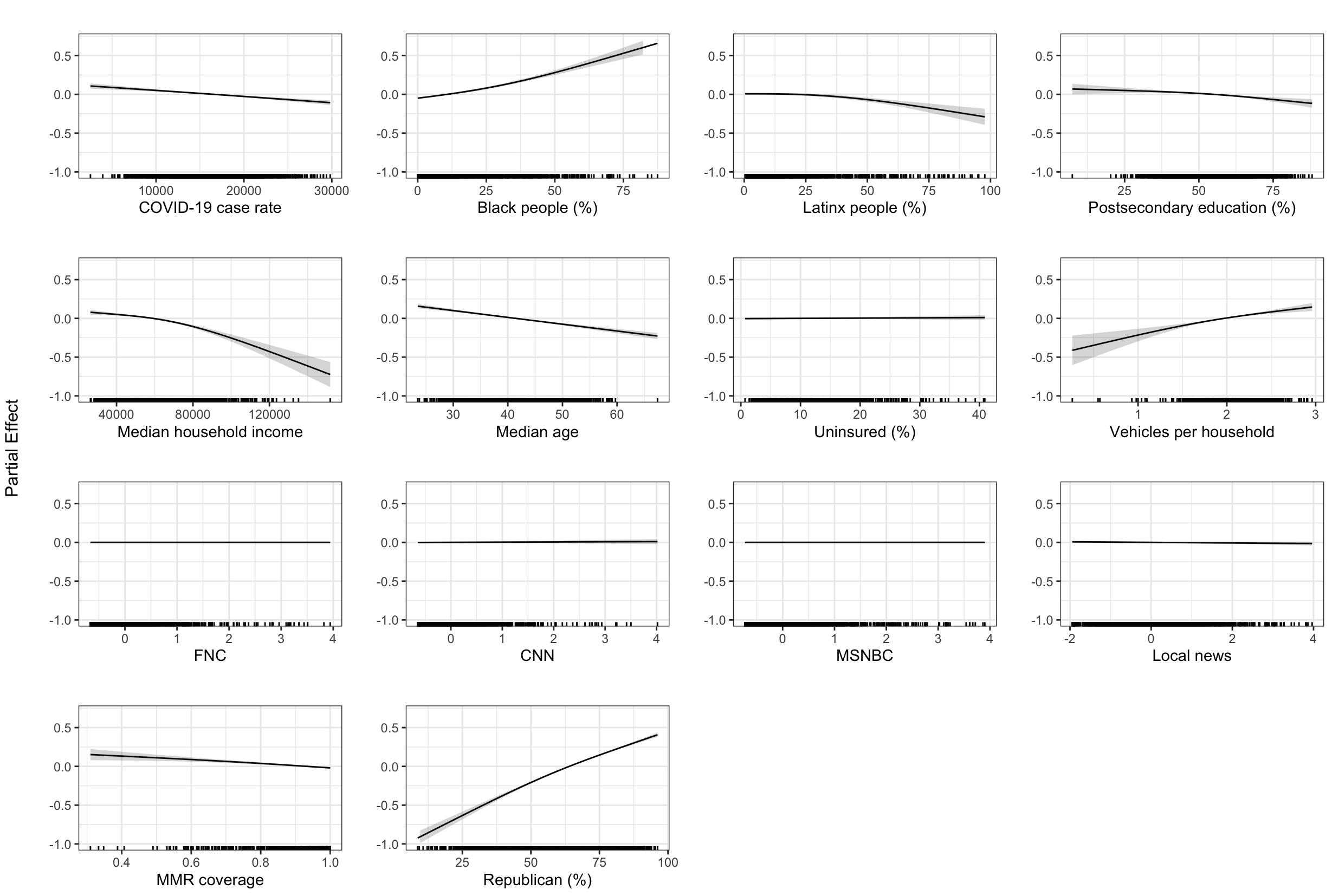


**Figure S4.** GAM results for the primary model with unified y-axis range. The shaded regions in each curve refer to the 95% confidence intervals, and the points at the bottom of each subplot indicates the distribution of each determinant.

| **Variables** | **Primary Model** | **Rural Counties** | **Urban Counties** | **Population Q1** | **Population Q2** | **Population Q3** | **Population Q4** | **Twitter Misinfo** |
| --- | --- | --- | --- | --- | --- | --- | --- | --- |
| COVID-19 case rate | < 2e-16 * | < 2e-16 * | 0.0422 * | < 2e-16 * | 1.34e-05 * | 0.00547 * | 0.008819 * | 0.11614 |
| Percentage of Black people | < 2e-16 * | < 2e-16 * | < 2e-16 * | < 2e-16 * | < 2e-16 * | < 2e-16 * | < 2e-16 * | < 2e-16 * |
| Percentage of Latinx people | < 2e-16 * | 0.00264 * | 4.14e-06 * | 0.033242 * | 0.001342 * | 3.63e-05 * | 0.000141 * | 1.5e-05 * |
| Postsecondary education | < 2e-16 * | < 2e-16 * | 0.02 * | 0.024678 * | 3.32e-06 * | 0.00404 * | 0.423629 | 0.00178 * |
| Median household income | < 2e-16 * | 2.17e-05 * | < 2e-16 * | 1.64e-06 * | 0.110638 | 3.73e-05 * | < 2e-16 * | < 2e-16 * |
| Median age | < 2e-16 * | < 2e-16 * | < 2e-16 * | 0.000939 * | 0.017459 * | < 2e-16 * | < 2e-16 * | < 2e-16 * |
| Uninsured percentage | 0.217 | 0.00114 * | 0.066 | 0.026748 * | 0.014393 * | 0.01699 * | 0.045869 * | 0.29917 |
| Vehicles per household | < 2e-16 * | < 2e-16 * | < 2e-16 * | 5.48e-07 * | 0.000404 * | 0.01160 * | < 2e-16 * | < 2e-16 * |
| Fox News viewership | 0.975 | 0.7621 | 0.2869 | 0.133697 | 0.389378 | 0.58551 | 0.804054 | 0.03507 * |
| CNN viewership | 0.236 | 0.48825 | 0.1758 | 0.578328 | 0.816158 | 0.54402 | 0.36291 | 0.77838 |
| MSNBC viewership | 0.454 | 0.15819 | 0.3012 | 0.377947 | 0.801062 | 0.00291 * | 0.769641 | 0.94142 |
| Local news viewership | 0.105 | 0.03535 * | 0.9412 | 0.187104 | 0.058216 | 0.56133 | 0.845971 | 0.14927 |
| MMR coverage | < 2e-16 * | 9.18e-07 * | 3.97e-05 * | 0.359801 | 7.92e-06 * | 4.12e-05 * | 3.05e-05 * | 8.8e-05 * |
| Republican presidential vote % | < 2e-16 * | < 2e-16 * | < 2e-16 * | < 2e-16 * | < 2e-16 * | < 2e-16 * | < 2e-16 * | < 2e-16 * |
| Twitter misinformation % | n/a | n/a | n/a | n/a | n/a | n/a | n/a | 0.09348 |

**Table S1.** P-values for significance of smooth terms in each GAM. Values less than 0.05 are marked with an asterisk (*).
